# Assessment of extracellular vesicle isolation methods from human stool supernatant

**DOI:** 10.1002/jev2.12208

**Published:** 2022-04-05

**Authors:** Emmalee J. Northrop‐Albrecht, William R. Taylor, Bing Q. Huang, John B. Kisiel, Fabrice Lucien

**Affiliations:** ^1^ Division of Gastroenterology and Hepatology Mayo Clinic Rochester Minnesota USA; ^2^ Microscopy and Cell Analysis Core Mayo Clinic Rochester Minnesota USA; ^3^ Department of Urology Mayo Clinic Rochester Minnesota USA

**Keywords:** biomarkers, cell‐derived microparticles, colonic neoplasms/diagnosis, extracellular vesicles/chemistry, gastrointestinal microbiome, tumour

## Abstract

Extracellular vesicles (EVs) are of growing interest due to their potential diagnostic, disease surveillance, and therapeutic applications. While several studies have evaluated EV isolation methods in various biofluids, there are few if any data on these techniques when applied to stool. The latter is an ideal biospecimen for studying EVs and colorectal cancer (CRC) because the release of tumour markers by luminal exfoliation into stool occurs earlier than vascular invasion. Since EV release is a conserved mechanism, bacteria in stool contribute to the overall EV population. In this study, we assessed five EV separation methods (ultracentrifugation [UC], precipitation [EQ‐O, EQ‐TC], size exclusion chromatography [SEC], and ultrafiltration [UF]) for total recovery, reproducibility, purity, RNA composition, and protein expression in stool supernatant. CD63, TSG101, and ompA proteins were present in EV fractions from all methods except UC. Human (18s) and bacterial (16s) rRNA was detected in stool EV preparations. Enzymatic treatment prior to extraction is necessary to avoid non‐vesicular RNA contamination. Ultrafiltration had the highest recovery, RNA, and protein yield. After assessing purity further, SEC was the isolation method of choice. These findings serve as the groundwork for future studies that use high throughput omics technologies to investigate the potential of stool‐derived EVs as a source for novel biomarkers for early CRC detection.

## 1 INTRODUCTION

Colorectal is the third most common cancer and ∼53,000 related deaths will occur this year in the United States (Siegel et al., 2021). Noninvasive screening for colorectal cancer (CRC) by the multitarget stool DNA (MT‐sDNA) test has revolutionized patient care. This test assays methylated *BMP3* & *NDRG4*, mutant *KRAS* and faecal haemoglobin. It has a specificity of 87%, and a sensitivity of 93% for stages I to III (Imperiale et al., 2014). However, the sensitivity for advanced precancerous lesions and nonadvanced adenomas is 42% and 17% (Imperiale et al., 2014). Therefore, there is a need to develop complementary biomarkers to augment the sensitivity and specificity of methylated DNA in order to identify patients with precancerous lesions at risk of developing CRC.

Extracellular vesicles (EVs) are microscopic particles (∼30 nm to 10 μm) abundantly released into body fluids by all types of cells including tumour cells. Tumour‐derived EVs contain cargo (RNA, proteins) that elicit various signalling pathways associated with cancer progression. These pathways involve: promoting cell proliferation and escape from apoptosis, sustaining angiogenesis, cell invasion and metastasis, reprogramming energy metabolism, transferring mutations, and modulating the tumour microenvironment by evading immune response and promoting inflammation (Xavier et al., 2020). Therefore, EV contents serve as potential biomarkers for diagnosis and disease monitoring because they provide a spatiotemporal fingerprint of the cell of origin and reflect the pathophysiological events occurring within the source tissue. Additionally, their role in mediating intracellular communication makes them ideal for natural drug delivery systems for anti‐cancer therapies.

High purity separation of EVs from interfering non‐vesicular components is a critical factor for biomarker discovery using ‐omic technologies. Ultracentrifugation is the most common method for EV separation; however, it is time consuming, labour intensive, and requires specific instrumentation. To address these challenges, several alternative techniques have recently been developed to isolate EVs from biofluids. Polymer‐based precipitation solutions such as ExoQuick (System Biosciences, Palo Alto, CA, USA) uses polyethylene glycol which forms a mesh like polymeric web that captures EVs and other contaminants of a certain size (usually 60–180 nm). Size exclusion chromatography separates particles based on their size as they pass through a column packed with a porous, polysaccharide resin. Fractions rich in EVs are then concentrated further by ultracentrifugation or ultrafiltration. Ultrafiltration uses a porous membrane to capture particles of a specific size and allows smaller particles to flow through the membranous filter. While many previous studies have investigated these EV isolation methods from a variety of biofluids, including blood (Barreiro et al., 2020; Brennan et al., 2020; Dhondt et al., 2020; Dong et al., 2020; Tian et al., 2020), data on the application of these techniques to stool supernatant are lacking.

Stool is the ideal biospecimen for studying EVs in association with CRC because the release of tumour markers by luminal exfoliation into stool occurs earlier than vascular invasion, hypothesized to be required for EV entry into the blood plasma compartment (Ahlquist, 2018; Ahlquist et al., 2012). Stool is composed of water, protein, undigested fats, polysaccharides, ash, undigested food residues, and a variety of bacteria (Rose et al., 2015). Specifically, the faecal microbiota contains diverse types of bacteria participating in immune protection of the gut, metabolism, and integrity of the intestinal epithelium. Recently, the microbiome has also been involved in CRC initiation and progression, and microbiota signatures have been linked to CRC development (Flemer et al., 2017; Ternes et al., 2020). EVs are released by all three domains of life (eukaryotes, bacteria, and archaea) and represent a universal, evolutionarily conserved mechanism (Gill et al., 2019). Surface antigens from donor cells allows for the enumeration and characterization of specific EV subpopulations by nanoscale flow cytometry and immunocapture, respectively (Brett et al., 2017; Gomes et al., 2018). Thus, allowing for separation of host‐derived and microbiome‐derived EVs to not only identify cancer biomarkers, but also provide novel biological insights in CRC pathogenesis.

As part of a larger effort to lay discovery of next‐generation biomarkers for the early detection of CRC, we aimed to compare ultracentrifugation, precipitation, size exclusion chromatography, and ultrafiltration for total particle recovery, reproducibility, purity, RNA composition, and protein expression in stool supernatant. Furthermore, we provide novel observations on the abundance and molecular composition of host and bacterial‐derived EVs in stool.

## 2 MATERIALS AND METHODS

**FIGURE 1:**
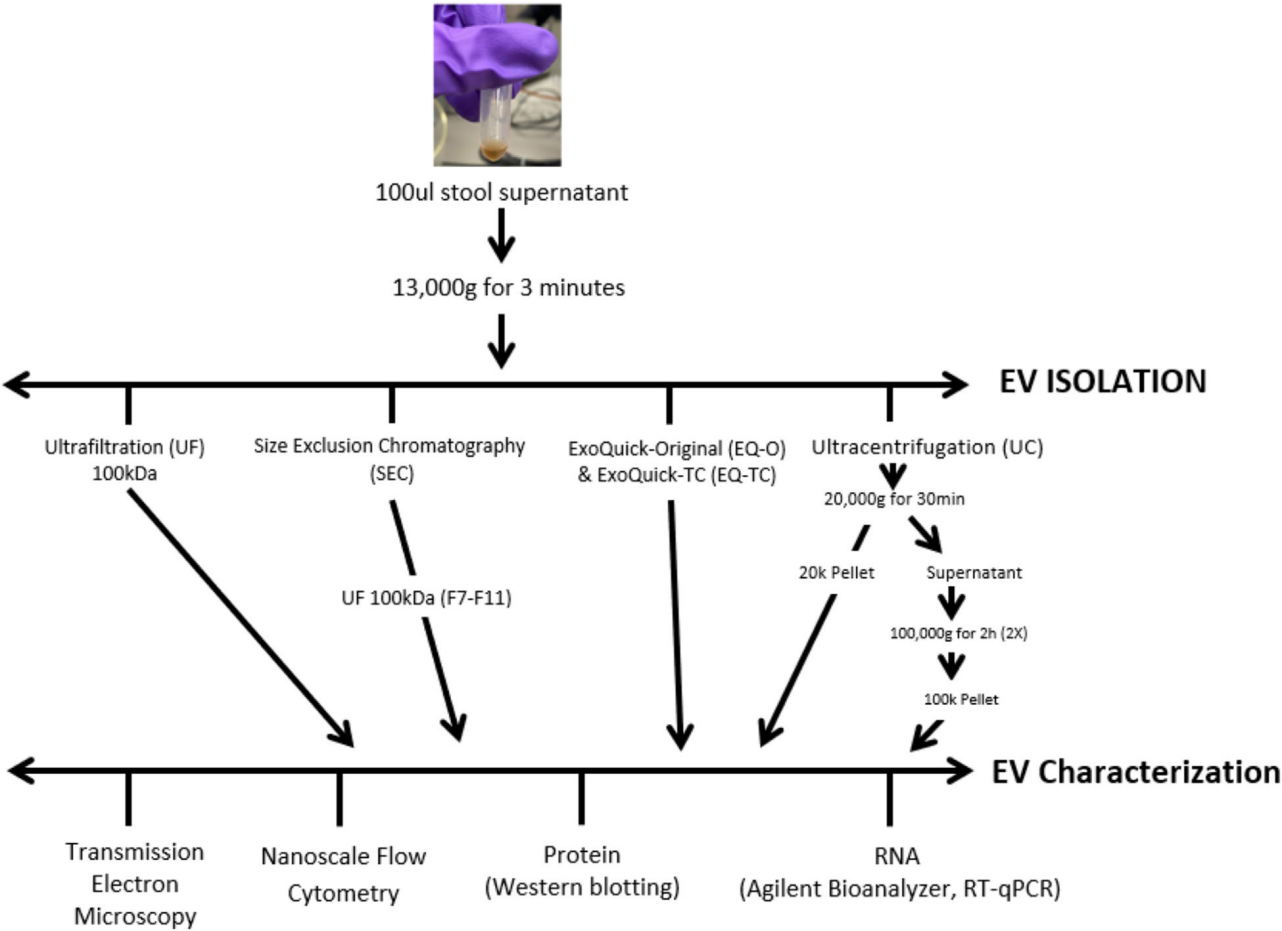
Schematic diagram of EV isolation/separation methods from human stool specimens. Extracellular vesicles were isolated from stool specimens using ExoQuick (original and TC), ultracentrifugation, ultrafiltration, and size exclusion chromatography. Several technologies were used in order to determine the most efficient isolation method (yield, purity) for downstream applications

The workflow followed in the present work is summarized in Figure 1.

### 2.1 Human specimens

Stool specimens from healthy individuals (*n* = 5) were obtained from an existing prospectively enrolled Institutional Review Board approved archive (IRB 18–008752) at Mayo Clinic Rochester Minnesota. Tris‐EDTA based preservative buffer was added to stool in a 1:5 ratio, aliquoted into multiple 50 ml volume conical tubes, and stored in a −80°C freezer until further processing for analysis. Samples were thawed overnight at 4°C. They were mixed by vortexing for 30 s, and then centrifuged at 4500 x *g* for 45 min. The supernatant was then transferred to a 50 ml conical tube. Stool supernatant (14 ml) was transferred to a 15 ml Falcon tube (Fischer Scientific, Waltham, MA, USA), and one polyvinylpolypyrrolidone (PVPP) tablet was added. The samples were then vortexed for 1 min and placed on a platform rocker for 15 min at room temperature. The mixture was transferred to a filter (8247 Frit) and centrifuged at 3300 x *g* for 6 min. The filtered and treated stool supernatant was stored at −80°C until isolation.

Platelet‐free plasma (PFP; *n* = 5) and cell‐free urine (urine; *n* = 5) samples were collected from benign individuals with approval from the Mayo Clinic Institutional Review Board (IRB 19–011292). Whole patient blood was drawn using a 21G needle in EDTA‐coated vacutainers (BD Biosciences, San Jose CA). Within 60 min post‐collection, vacutainers were centrifuged at 2500 x *g* for 15 min at 20°C, and plasma was transferred to 15 ml conical centrifugal tubes without disturbing the pellet. The plasma was centrifuged again at 2500 x *g* at 20°C for 15 min. The top layer of plasma was transferred to cryovials and stored at −80°C until further use. First‐catch urine was collected using a Colli‐Pee collection device (Novosanis, Wijnegem, Belgium). Within 10 min post‐collection, urine was transferred to 50 ml conical tubes (Fischer Scientific, Waltham MA) and centrifuged at 3000 x *g* at 20°C for 15 min. Supernatant was transferred to cryovials and stored at −80°C until further analysis.

### 2.2 Isolation of EVs

EVs were isolated from 100 μl and 500 μl of stool supernatant by five separation methods including four commercially available products and conventional ultracentrifugation. Manufacturer's instructions were followed for each method. Stool supernatant was centrifuged at 13,000 x *g* for 3 min prior to isolation to remove any cellular debris that was not removed during processing. When using 500 μl of stool supernatant, EV isolation systems were saturated based on poor recovery rates. Therefore, this manuscript focuses on data collected from 100 μl of stool supernatant.

#### 2.2.1 Ultracentrifugation (UC)

Stool supernatant was thawed and centrifuged at 20,000 x *g* for 30 min at 4°C in a Thermo Scientific (Waltham MA) Sorvall Legend Micro 17R centrifuge. The pellet was suspended in 100 μl of PBS (UC‐P20k). The supernatant was diluted in 5 ml of PBS and centrifuged at 100,000 x *g* for 2 h at 4°C in a Beckman Coulter (Brea CA) Optima XPN‐100 ultracentrifuge using a SW 55 Ti rotor. The pellet was washed with 5 ml of PBS and was followed by a second UC at 100,000 x *g* for 2 h at 4°C. The pelleted EVs (UC‐P100k) were resuspended in 200 μl of PBS. We tested several UC protocols at varying speeds and times and determined the method above resulted in the purest stool‐derived EVs based on TEM with median recovery at 31% (Figures S1 and S2).

#### 2.2.2 Original ExoQuick isolation reagent (EQ‐O)

The Original ExoQuick (System Biosciences, Palo Alto, CA, USA) was used in the current study; 25.2 μl of reagent was added to 100 μl of stool supernatant and mixed by inverting tube. The samples were incubated overnight at 4°C, and centrifuged at 1500 x *g* for 30 min at 4°C. The supernatant was removed, and samples were centrifuged at 1000 x *g* for 2 min to remove any residual fluid. The supernatant was removed and stored for nFCM analysis (F1). The pellet (P1) was resuspended in 100 μl of PBS.

#### 2.2.3 ExoQuick Tissue Culture media isolation reagent (EQ‐TC)

ExoQuick‐TC (System Biosciences, Palo Alto, CA, USA) was also used in the current study; 20 μl of reagent was added to 100 μl of stool supernatant and mixed by inverting tube. The samples were incubated overnight at 4°C, and centrifuged at 1500 x *g* for 30 min at 4°C. The supernatant was removed, and samples were centrifuged at 1500 x *g* for 5 min to remove any residual fluid. The supernatant was stored for nFCM analysis(F1), and the pellet (P1) was resuspended in 100 μl of PBS.

#### 2.2.4 Ultrafiltration (UF)

Amicon ultra‐0.5 centrifugal filter devices (Millipore Sigma, Amicon Ultra 100k device, Burlington, MA, USA) were used to isolate and concentrate EVs from stool supernatant. The 100 μl of stool supernatant was further diluted by adding 400 μl of PBS and was transferred into the filter device. Samples were centrifuged at 14,000 x *g* for approximately 10 min at 4°C. The flow through was kept for nFCM analysis. The filter containing the concentrate was then turned upside down into a new tube and centrifuged at 1000 x *g* for 2 min, and the volume was recorded.

#### 2.2.5 qEV column (SEC)

The qEV columns (qEVsingle < 150 μl /70 nm, IZON Science, Christchurch, NZ) were equilibrated with 5 ml of PBS prior to using. Then, 100 μl of stool supernatant was pipetted onto the column and collected into a tube. Each subsequent fraction (200 μl) was collected in a separate tube. After nFCM analysis, fractions rich in EVs (7‐11) were combined, and further concentrated by ultrafiltration using Amicon ultra‐4 ml 100 kDa centrifugal filter devices.

### 2.3 Nanoscale Flow Cytometry (nFCM)

All samples were analysed by an A60‐MicroPlus nanoscale flow cytometer (Apogee Flow Systems Inc., Hertfordshire UK). Before sample analysis, the A60‐MicroPlus was calibrated using a Rosetta calibration bead mix (Exometry Inc., Amsterdam, Netherlands) as previously described (van der Pol et al., 2018). Side scatter triggering threshold was set at 1700 a.u corresponding to a scattering cross‐section of 12 nm^2^ and a particle diameter of 168 nm (Refractive Index core = 1.38; Refractive Index shell = 1.48; shell thickness = 4 nanometres). To determine percentage recovery, samples were diluted in sterile PBS and were ran at a flow rate of 0.75 μl/min for 1 min with an event rate below 7000 events per second to avoid swarm effect. Before each run, nFCM underwent a quality control procedure including a run with a mix of polystyrene and silica polydisperse beads (Apogee bead mix #1493, Apogee Flow Systems) to control for instrument sensitivity and flow rate stability. Buffer‐only control (sterile PBS) was analysed with the same instrument/acquisition settings and the event rate was kept below 80 events per second. To measure ApoB^+^ particles, stool supernatant samples were incubated with ApoB‐PE antibody (clone A‐6, sc‐393636) at a final concentration of 35 μg/ml for 30 min in the dark. The count rate with antibody‐only control (anti‐ApoB in PBS) was below 11 PE^+^‐events in 1 min. Each sample was run in duplicate. Particle concentration and sample volume were used to calculate total particle count in each sample. Quantification of total particles and particle diameter was performed using FlowJo v10.6.1 software (FlowJo LLC., Ashland, OR, USA). 

### 2.4 Transmission electron microscopy

Transmission electron microscopy (TEM) was carried out at Mayo Clinic's Microscopy and Cell Analysis Core. Extracellular vesicles were fixed in an equal volume of 4% paraformaldehyde /0.1 M phosphate buffer overnight at 4°C. Sample (5 μl) was placed on a formvar‐carbon‐coated nickel grid (200 mesh), air dried for 30 min, and washed with PBS (6 × 3 min). The samples were then fixed in 1% glutaraldehyde/PBS for 5 min and washed with distilled water (6 × 3 min). They were transferred to a mixture of 4% uranyl acetate (freshly made) and 2% methylcellulose (in 1:9 ratio) for 5 min. Filter paper was used to soak up the solution that was left on the formvar coated grids and they were air dried for 1 h. Samples were observed with a JEOL (Peabody MA) 1400 electron microscope. Diameter of EVs was measured manually using Fiji software. EVs on the edge of the image or folded were excluded from the analysis. A total of 54–109 vesicles were measured per method for a single patient.

For immunogold staining, grids were blocked with 10% FBS for 20 min followed by 1 h incubation at room temperature with a primary antibody (CD63 rabbit polyclonal or OMPA rabbit polyclonal antibody) or diluted in 1:20 ratio in blocking solution. Next, grids were incubated with donkey antirabbit IgG (H + L) conjugated with 6 nm gold particles (Jackson ImmunoResearch, West Grove PA) for 1 h, rinsed with PBS, fixed in 1% glutaraldehyde for 5 min and washed with distilled water. The grids were then contrasted and embedded in a mixture of 4% uranyl acetate and 2% methylcellulose in 1:9 ratio. The grids were examined with a JEOL 1400 electron microscope at 80 kV.

### 2.5 Protein concentration assay (BCA)

Extracellular vesicles samples were incubated with Pierce RIPA buffer and Halt protease inhibitor cocktail (Thermo Scientific, Waltham, MA) at 4°C for 30 min. Samples were then centrifuged at 13,000 x *g* for 5 min at 4°C, and supernatant containing protein lysates were aspirated. Protein concentrations were determined by Pierce BCA Protein Assay Kit (Thermo Scientific, Waltham, MA) following the manufacturer's instructions.

### 2.6 Western blot

NuPAGE LDS sample buffer (4x) and NuPAGE reducing agent (10x) (Fischer Scientific, Waltham MA) were added to equal protein amounts from each of the EV isolation methods and stool supernatant. Samples were run on NuPAGE 4–12%, Bis Tris, 1.0 m, mini protein gel (Fischer Scientific, Waltham, MA, USA) after boiling for 10 min at 70°C. Proteins were transferred to nitrocellulose membranes using the iBlot 2 transfer device and were blocked with 5% BSA for 30 min. The membranes were then incubated overnight at 4°C with the following antibodies: groEL (1:1000, abcam, ab90522), TSG101 (1:1000, abcam, ab30871), ApoB (1:1000, Santa Cruz Biotechnology, sc‐393636), CD63 (1/1000, abcam, ab231975), ompA (1/5000, Antibody Research, 111120), Flagellin (1/10000, abcam, ab93713), Calnexin (1/1000, abcam, ab22595), HSP70 (1/1000, System Biosciences, Exoab‐hsp70A‐1), and LPS (1/1000, abcam, ab35654). After incubation with primary antibodies, the membranes were washed with 0.1% tween in tris‐buffered saline (TBST) three times for 5 min each. Incubation with secondary antibody was performed at room temperature with goat anti‐rabbit or anti‐mouse IgG secondary antibody, HRP conjugated (1:10000; Invitrogen, Waltham MA) for 1 h. Membranes were washed with 0.1% tween in tris‐buffered saline (TBST) three times for 10 min each. Membranes were exposed to Supersignal West Pico Plus chemiluminescent substrates (Fischer Scientific, Waltham MA) in 1:1 ratio and incubated for 5 min prior to imaging on a Chemidoc XRS+ system (Bio‐Rad Laboratories, Hercules CA). If proteins overlapped in size, Restore Western Blot Stripping Buffer (Thermo Scientific, Waltham, MA) was used to strip antibodies from the membranes prior to reblotting.

### 2.7 RNA extraction and RT‐qPCR

RNA was extracted from EVs using the Qiagen miRNeasy Mini Kit (Germantown MD) following the manufacturer's instructions. The on‐column DNase digestion with the RNase‐Free DNase Set was performed. Each RNA sample (1 μl) was loaded on a RNA 6000 Pico chip (Agilent Technologies, Santa Clara CA) to determine concentration and fragment size distribution.

Total cellular RNA from the five samples and four isolation methods (UC, EQ‐O, SEC, UF) were pooled. Concentration was determined using the Quant‐iT RiboGreen RNA Assay Kit (Invitrogen, Waltham MA). RNA was reverse transcribed into cDNA using the iScript cDNA synthesis kit (Bio‐Rad, Hercules CA). IDT PrimeTime qPCR primers/probes (Table 1) were designed for 18s rRNA (human) and 16s rRNA (bacteria) and diluted to 10μM. The 16s primers/probe set was designed based on conserved regions among prevalent bacteria *(Eubacterium rectale, Bacteroides vulgatus, and Faecalibacterium prausnitzii)* in stool. Primer efficiencies were determined based on a standard curve generated from a 2‐fold serial dilution. RT‐qPCR (8 ng) was performed in duplicate using PrimeTime Gene Expression Mastermix (IDT, Coralville IA) on a LightCycler 480 Instrument (Roche Diagnostics, Indianapolis IN). A no template control was present on the plate to assure no background contamination. The PCR program was 3 min at 95°C for polymerase activation, 95°C for 15 s for denaturation followed by annealing/extension for 1 min at the given annealing temperature (Table 1). The ratio of EV RNA from bacteria and human was adjusted according to primer efficiencies followed by the 2^∆Ct^ method.

**TABLE 1:** RT‐qPCR primer and probe sequences for 16s rRNA (bacteria) and 18s rRNA (human)

Target	Amplicon size	Tm	Forward primer	Probe	Reverse primer
16s rRNA	140 bp	44C	GCGGTGAATACGTTCCCGGG	TGTACACACCGCCCGTCA	TACCTTGTTACGACTT
18s rRNA	82 bp	49C	CACGGACAGGATTGACAGATT	AGCTCTTTCTCGATTCCGTGGGTG	ATCGCTCCACCAACTAAGAAC

### 2.8 Enzymatic treatment

Extracellular vesicles from UF and SEC were treated with proteinase K (Thermo Scientific, Waltham MA) for a final concentration of 500 ng/μl for 30 min at 37°C. Proteinase K was inactivated by incubating the samples for 10 min at 90°C. Then, the samples were treated with RNase A (Thermo Scientific, Waltham, MA, USA) at a final concentration of 10 ng/μl for 15 min at 37°C. Total RNA was then extracted as described above. Concentration and fragment sizes were analysed on an Agilent RNA 6000 Pico and Small RNA chip.

## 3 RESULTS

### 3.1 Electron microscopy reveals purity and size of EVs from stool specimens

**FIGURE 2:**
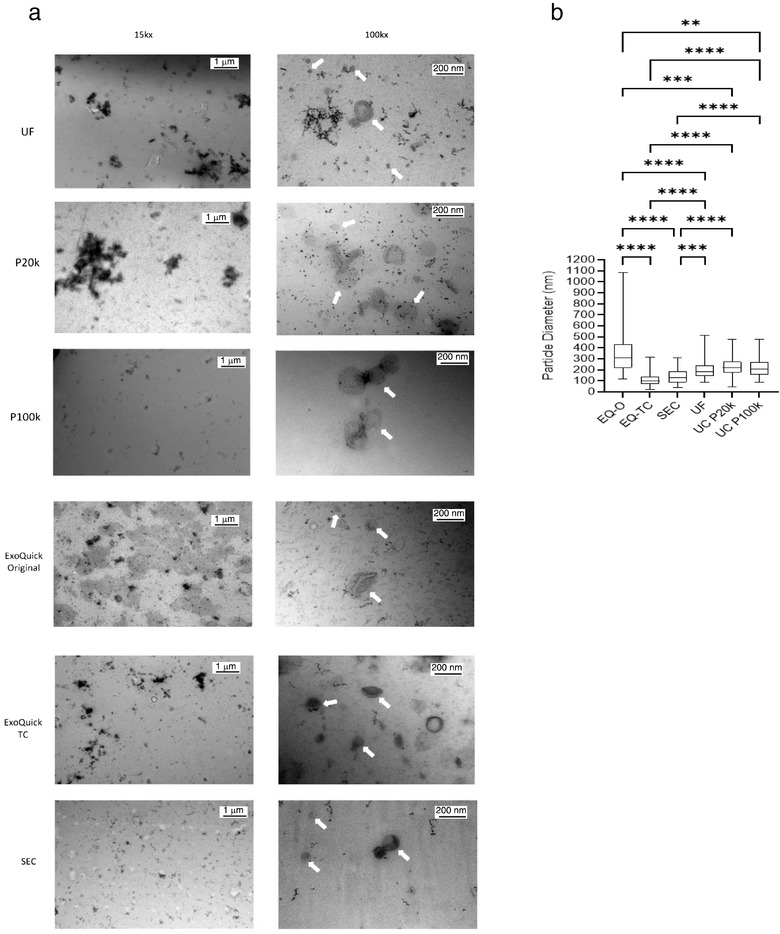
Assessment of purity of EV preparations from human stool specimens by electron microscopy. (a) Representative EM pictures showing EVs isolated from five different methods with UC‐P20k and UC‐P100k captured separately at 15kx and 100kx. Lower magnification shows a wider field of view and a better representation of contaminants in samples. White arrows at higher magnification indicate individual EVs. No lipoprotein contamination was observed with any of the methods (b) Box and whisker plot depicting the size of vesicles (nm) as measured by TEM images, there were 54–109 vesicles counted per method, Kruskal‐Wallis test, ****p < 0.0001, ***p < 0.0005, **p < 0.01

By using electron microscopy, the presence of intact cup‐shaped EVs after isolation was confirmed in all five methods. Many of the methods had visible contaminants, but ultracentrifugation (P100k) and SEC appeared to have the least (Figure 2a). We did not observe lipoprotein particles in EV samples analysed by electron microscopy. This is in line with nFCM quantification of particles positive for the lipoprotein marker, Apolipoprotein B (ApoB). Given that platelet‐free plasma is rich in lipoproteins, we compared levels of ApoB^+^ particles in plasma (*n* = 5) and stool (*n* = 5) samples. We measured 36.32% and 0.73% ApoB^+^ particles in plasma and stool suggesting that lipoproteins are not a major contaminant in stool (Figure S3A). Additionally, ApoB was not detected by western blot in crude stool supernatant and purified EVs isolated from stool (Figure S3B).

Vesicle diameters for each method (*n* = 54–109 vesicles) were measured using transmission electron microscopy images (Figure 2b). ExoQuick‐ original enriched for larger vesicles with the median vesicle size of 307 nm with a lot of variability within the group (Min:121 nm, Max: 1085 nm). ExoQuick‐TC enriched for smaller vesicles with the median vesicle size of 99 nm with the least variability (Min:24 nm, Max: 315 nm). For ultracentrifugation, there was not a significant difference in median particle size among the vesicles isolated from the 20k and 100k centrifugation (220 nm vs. 208 nm). Ultrafiltration and SEC had median particle sizes of 181 nm and 133 nm.

### 3.2 Size‐exclusion chromatography and ultrafiltration provide higher particle recovery from human stool specimens

**FIGURE 3:**
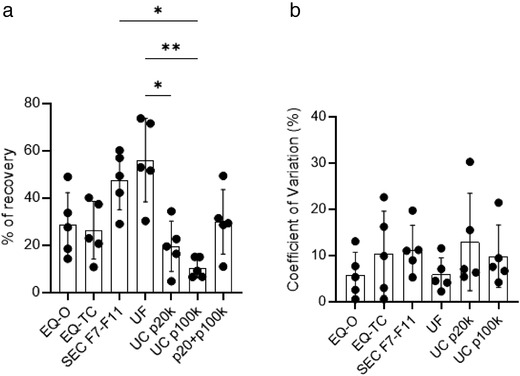
nFCM‐based comparative analysis of EV isolation/enrichment methods for particle recovery from five healthy human stool specimens. (a) total particle recovery for the five isolation methods, with ultracentrifugation further broken down into P20k and P100k, Mean ± SEM, Friedman test, *p < 0.05, **p < 0.01 (b) Coefficient of variation for recovery within each patient and method, Mean ± SEM, Friedman test, there was no significance present. Source of variation in recovery is likely due to differences in the composition of stool specimens

The A60‐Micro Plus nanoscale flow cytometer (nFCM) can resolve polystyrene and silica beads in a size range from 80 nm to 1300 nm (Figure S3C). To estimate diameter of stool‐derived particles, we used scatter‐to‐ratio relationship described by Mie theory (van der Pol et al., 2018). A mixture of NIST certified and traceable polystyrene beads (Rosetta beads) with known diameters and refractive indexes were acquired, and side scatter intensities of each bead population were converted to nanometres. For this study, the acquisition settings allowed for detection of a polystyrene bead population with a diameter of 80 nm and a refractive index (RI) of 1.62 at 405nm wavelength which scatter as much light as an EV of 158 nm +/‐ 10 nm (RI core = 1.38; RI shell = 1.48; shell thickness = 4 nm) (van der Pol et al., 2018) (Figure S3D). To measure recovery efficiency of EV isolation/enrichment methods, we compared particle numbers from five stool specimens pre‐ and post‐isolation/enrichment (Figure S4). We observed that ultrafiltration (UF) and size‐exclusion chromatography (SEC) were the most efficient methods to recover particles with a median recovery rate of 53% and 49%, respectively, (Figure 3a). ExoQuick‐TC and ExoQuick‐Original were the least efficient enrichment methods with a median recovery rate of 23% and 28%. For ultracentrifugation, we carried out several protocols altering the time and/or speed of the spins to optimize recovery and purity of EVs in stool specimens (Figures S1, S2). While other protocols we tested yielded better recovery, they appeared to have more visible contaminants by TEM. For the chosen protocol we measured particle number in pellets obtained after 20,000 x *g* (P20k) and 100,000 x *g* (P100k) centrifugation. In four out of five samples, higher particle count was obtained in the P20k fraction compared to the P100k fraction (Figure S4). Median particle recovery rate was 19% and 9% for P20k and P100k, respectively (Figure 3a).

To assess the reproducibility of EV isolation methods, we performed each method in three technical replicates for each specimen and calculated the coefficient of variation in particle recovery (Figure 3b). High reproducibility (%CV < 13%) was observed across all methods with UF performing the best with a median (%CV± SD) of 4.5 ± 3.6 and SEC performing the worst with 11.0 ± 5.3. This indicates that the source of variation in recovery data is due to differences in the composition of stool specimens among the patients (Figure 3b, Figure S5).

To determine whether EV isolation methods performed similarly with a larger sample volume, we also measured particle recovery from 500 μl of stool supernatant which is five times more than the volume used above (Figure S6A). Median recovery rate was 47.7% for SEC, followed by 30.8% for UC (P20k+P100k) and 26.0% for EQ‐O. Ultrafiltration was inefficient using 500 μl of stool samples with a median recovery rate of 0.025%, which may be caused by a damaged filter and particle loss due to high particle concentration and system saturation. Overall, all methods performed worse with 500 μl of initial sample. We also established that stool supernatant has high particle concentrations using nFCM compared to other biofluids such as platelet‐free plasma and cell‐free urine (Figure S6B).

### 3.3 Bacteria and host‐derived EVenriched protein markers are detected in human stool specimens

**FIGURE 4:**
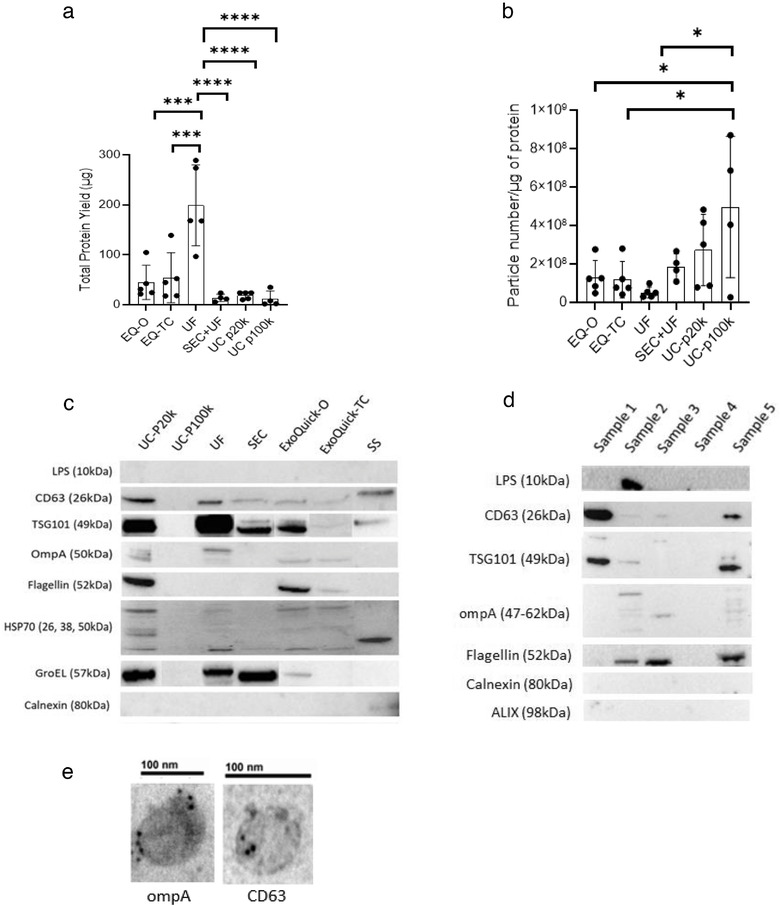
Characterization of protein content from stool‐derived EVs. (a) Total protein yield (μg) across the different EV isolation/enrichment methods measured by BCA assay, (*n* = 4–5 samples), Mean ± SEM, One‐way ANOVA test, ***p < 0.0005, ****p < 0.0001 (b) Ratio of total particle number and total protein yield, a higher ratio suggests less co‐isolated soluble protein contamination (*n* = 4–5 samples), Mean ± SEM, One‐way ANOVA test, *p < 0.05, (c) Western blot (8ug) showing human, bacterial, and contaminate protein expression post‐isolation for the five methods and pre‐isolation (stool supernatant) (d) Western blot (15ug) showing human, bacterial, and contaminate protein expression from five SEC EV samples (e) Immunogold labelled stool‐derived EVs (ompA, CD63), magnification: 150kx, 200kx

We measured protein yield in stool samples following EV isolation/enrichment methods. Protein yield from EV fractions were (mean ±SD; μg): 199.12 ± 80.69 (UF), 13.75 ± 7.47 (SEC), 44.84 ± 34.33 (EQ‐O), 54.28 ± 49.82 (EQ‐TC), 17.53 ±7.40 (UC‐P20k), and 11.62 ± 15.76 (UC‐P100k) (Figure 4a). The amount of non‐EV protein contamination present was determined by calculating the ratio of particle count and protein yield (Webber & Clayton, 2013) (Figure 4b). Ultracentrifugation and SEC yielded higher particle to protein ratios indicating less coisolated non‐vesicular soluble proteins present compared to the other isolation methods.

To further characterize stool‐derived EVs from the different isolation methods, we analysed the expression of human, bacterial, and contamination proteins by western blot (Figure 4c, 4d) and immunogold labelling (Figure 4e). To examine the presence of contaminants, we selected calnexin and flagellin (Théry et al., 2018; Tulkens et al., 2020). Calnexin was only detected in stool supernatant, which indicates there is no protein contamination from endoplasmic reticulum compartments in EV fractions from all methods. Flagellin has been identified in Gram negative bacterial EVs as a major subunit of flagella (Lee et al., 2016) and a potential contaminant in stool‐derived EV preparation (Klimentová & Stulík, 2015). It was detected in the UC‐P20k, EQ‐O, and EQ‐TC which may indicate soluble protein contamination. Additionally, it was also detected in SEC when protein input was increased (15 μg) (Figure 4d). Tetraspanins are typically enriched on the membranes of EVs, CD63 was detected in stool supernatant and all isolation methods except UC‐P100k. It was further confirmed by immunogold labelling of EVs isolated by SEC (Figure 4e). Additionally, HSP70, a cytosolic protein within EVs, was also present in all samples except UC‐P100k. For bacterial derived EV protein markers, we selected LPS and ompA as reported previously (Tulkens et al., 2020). For ompA, bands were present in all methods except UC‐P100k, while LPS was detected in one SEC sample when protein input was increased. We also analysed the expression of GroEL, a cytoplasmic molecular chaperone and the bacterial counterpart of human Hsp60 protein. GroEL has been previously found within bacteria derived EVs (Alkandari et al., 2020; Hong et al., 2019; Piotrowska et al., 2020). Recent research has indicated that it may be more of a contaminant since it is present in crude bacterial preparations and can be removed with further purification (Hong et al., 2019). TSG101, a human exosomal protein (Théry et al., 2018) was detected in EV samples isolated by all methods except UC‐P100k. Compared to crude stool supernatant, TSG101 was enriched in post‐isolation samples suggesting successful EV enrichment.

### 3.4 EV isolation/enrichment methods influence RNA yield and composition from human stool

**FIGURE 5:**
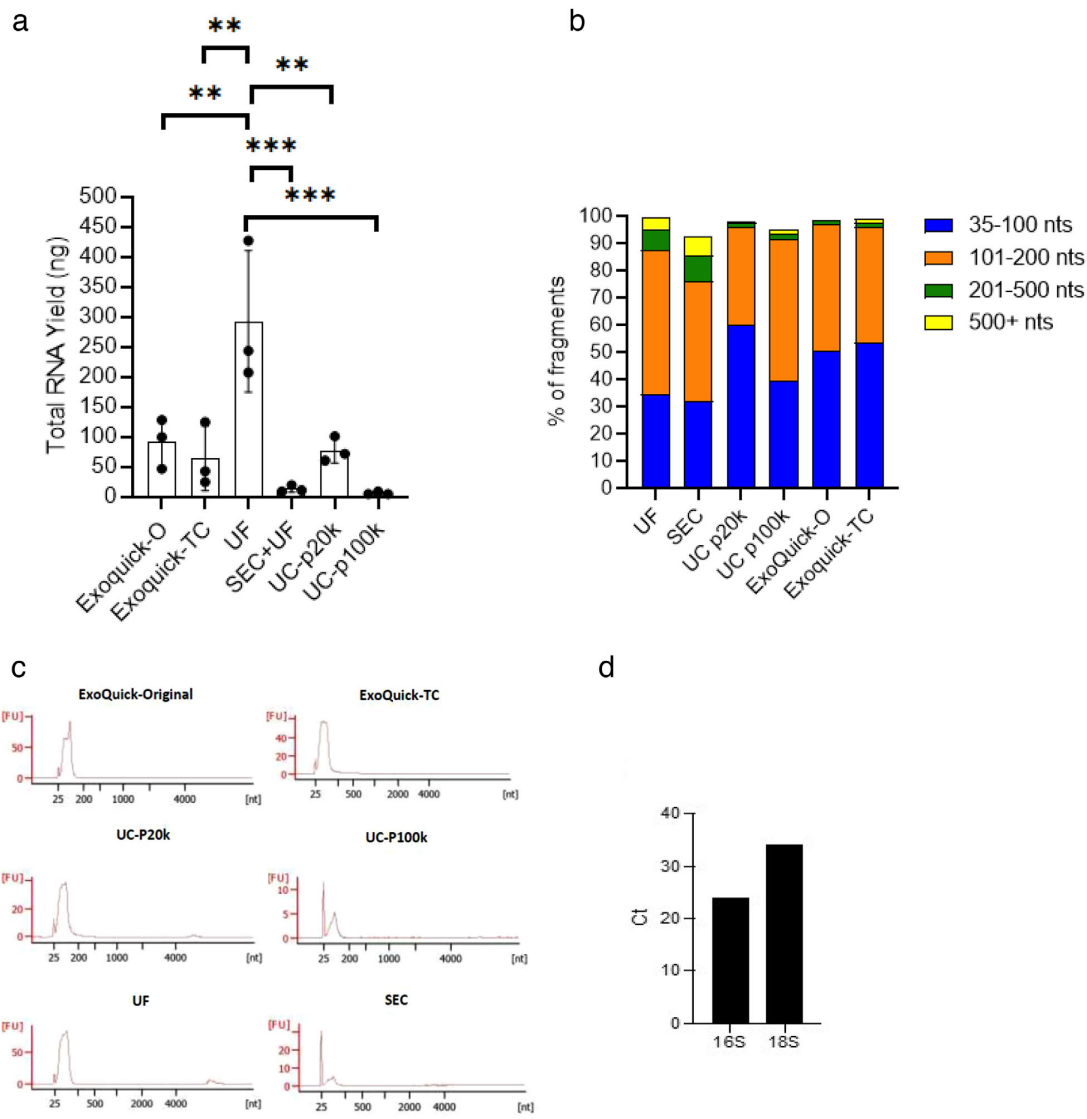
Characterization of RNA content from stool‐derived EVs with no prior enzymatic treatment. (a) Total RNA yield (ng) measured by the Agilent bioanalyzer Pico chip, Mean ± SEM, One‐way ANOVA test, **p < 0.001, ***p < 0.0005, (b, c) Bar chart and electropherograms depicting RNA fragment size distributions from three healthy patients (average) measured by the Agilent bioanalyzer Pico chip (d) RNA was extracted and pooled from EV fractions from four isolation methods and Ct (cycle threshold) values for 16s (bacteria) and 18s (human) rRNA were generated by RT‐qPCR. The lower Ct value indicates more bacterial rRNA is present in stool compared to host derived rRNA

Despite using a similar initial volume of stool supernatant, a significant difference was observed in RNA yield (mean ± SD; ng) among the different EV isolation/enrichment methods (Figure 5a). Ultrafiltration had the highest RNA yield (292.36 ± 117.99) followed by EQ‐O (91.3 ± 41.3), UC‐P20k (77.35 ± 20.93), EQ‐TC (63.68 ± 53.46), SEC (12.97±5.75), and UC‐P100k (5.70 ± 2.29). Fragment size distribution analysis revealed that EQ‐O had the largest proportion of fragments between 35 and 100 nt (62.3% ± 8.1), and SEC had the smallest proportion (39.3% ± 8.1) of fragments in this size range (Figures 5b, 5c). Ultracentrifugation‐P20k pellet had the largest proportion of fragments between 101 and 200 nt (48.0% ± 7.8), EQ‐O had the smallest proportion (37.0% ± 8.7) of fragments in this size range. Size exclusion chromatography had the largest proportion of fragments greater than 201 nucleotides (8.9%). To determine the presence of bacteria‐ and host‐derived RNA molecules, we measured the expression of 16S (bacteria) and 18S (human) ribosomal subunit transcripts by RT‐qPCR (Figure 5d). The 16S rRNA transcript was 1097x more abundant than 18S rRNA in the pooled EV fraction. For crude stool supernatant total RNA, 50x the input was necessary for later amplification of 16s rRNA (ct = 29). Various inputs of RNA from stool supernatant up to 1 μg resulted in no amplification of 18s rRNA. Overall, both bacterial and host‐derived RNA and proteins were detected in EV samples from stool specimens.

**FIGURE 6:**
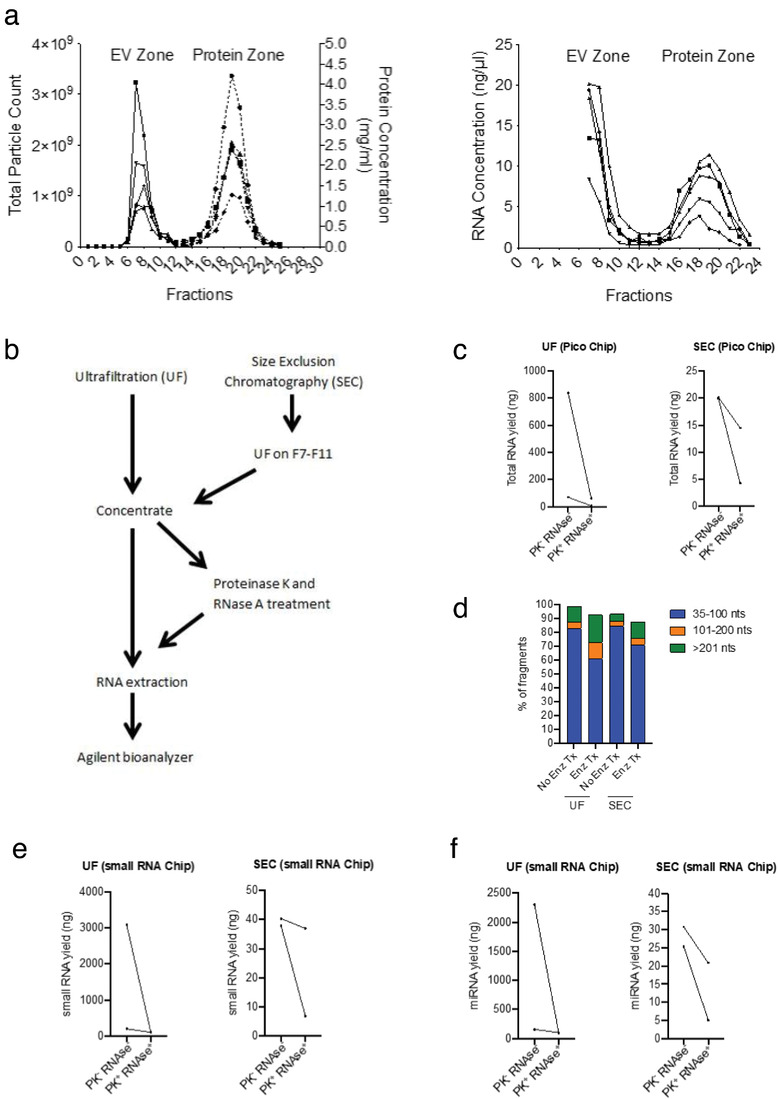
Assessment of RNA yield and composition after proteinase k and RNase A enzymatic treatment. (a) The first graph is the elution profile for the qEV‐70 single SEC column. The graph on the right shows increased RNA concentrations (Ribogreen) in both the EV and protein zones (b) Schematic workflow for determining the extent of non‐vesicular RNA contamination following UF and SEC (c) Total RNA yield (ng) measured by Agilent bioanalyzer Pico chip (*n* = 2 samples) (d) Bar graph showing fragment size distributions from two healthy patients (average) measured by the Agilent bioanalyzer Pico chip (e) small RNA and (f) miRNA yield measured by Agilent bioanalyzer small RNA chip (*n* = 2 samples)

Co‐isolation of non‐vesicular extracellular RNA is an important confounding factor affecting downstream RNA analysis (Mateescu et al., 2017). For instance, by using Ribogreen, we detected RNA in both EV and soluble protein fractions from stool samples processed by SEC (Figure 6a). Non‐vesicular RNA can bind to ribonucleoprotein complexes (RNPs) and lipoproteins (Jeppesen et al., 2019). To determine the presence of RNA outside of vesicles, we treated EVs from what appeared to be the most contaminated (UF) and least contaminated (SEC) methods with Proteinase K and RNAse A to disrupt RNPs (Figure 6b). Then, RNA yield and fragment size were measured by the Agilent Bioanalyzer Pico 6000 chip (Figures 6c, 6d). For samples that had no enzymatic treatment prior to extraction, the RNA yield for UF concentrate was 837.6 ng (Sample 1) and 70.8 ng (Sample 2). Following enzymatic treatment, RNA yield dropped to 63.0 ng and 7.6 ng for Sample 1 and 2, respectively. This corresponded to an RNA loss of 92.5% (Sample 1) and 89.3% (Sample 2). Samples processed with SEC showed an initial concentration of 19.9 ng (Sample 1) and 20.2 ng (Sample 2). Samples that were treated with proteinase k and RNase A had an RNA yield of 4.2 ng (Sample 1) and 14.5 ng (Sample 2) corresponding to an RNA loss of 78.7% (Sample 1) and 28.1% (Sample 2). These data shows that UF has more non‐vesicular RNA contamination than SEC. An average of approximately 9.1% of the isolated RNA is found within the vesicles for UF, while approximately 53.4% of the isolated RNA is from within the vesicles for SEC. Electropherograms for samples that did and did not undergo enzymatic treatment are depicted in Figure S7. The majority of the fragments were less than 100 nucleotides for both UF and SEC, regardless of enzymatic treatment exposure (Figure 6d). RNA samples were also analysed on an Agilent small RNA chip in order to further investigate the fragment size distribution for smaller sized fragments since they make up a large portion of the overall RNA (Figures 6e, 6f). This analysis revealed that UF samples have higher concentrations of small RNA and miRNA compared to SEC samples, and an average of 97.5% of the small RNA and 97% of miRNA was non‐vesicular in origin. For SEC samples, an average of 53% of the small RNA and 45% of the miRNA was from within the vesicles. Altogether, our findings demonstrate that SEC is a superior EV isolation method compared to UF in terms of RNA purity. Nevertheless, a large fraction of RNA is present in the form of non‐vesicular RNA and enzymatic treatment with proteinase k and RNase A is critical to minimize contamination in downstream EV‐RNA profiling.

## 4 DISCUSSION

The multitarget stool DNA (MT‐sDNA) test is a noninvasive screening option with a high sensitivity (93%; stages I‐III) for the detection of colorectal cancer (CRC) (Imperiale et al., 2014); however, the sensitivity for detecting advanced precancerous lesions remains under 50%. If detected early, CRC has a 5‐year survival rate around 90% for individuals with localized lesions (Siegel et al., 2021). For this reason, our group is interested in further investigating complementary biomarkers that augment the sensitivity of methylated DNA markers and improve the detection of advanced precancerous lesions (advanced adenomas and sessile serrated polyps ≥ 1 cm) before they become cancerous. Tumour derived EVs are an ideal source for potential biomarkers because their contents (nucleic acids, proteins, lipids) mirror molecular signatures from the cell of origin and the lipid bilayer protects the cargo from enzymatic degradation. However, a major challenge associated with EV research is the lack of standardized protocols for EV separation, cargo isolation, and characterization. To date, there is no gold standard for EV isolation from biofluids and the method of choice should be carefully selected based on the downstream application. Each EV isolation method has limitations in terms of yield and purity that can lead to cofounding factors in ‐omics related studies (Théry et al., 2018). To our knowledge, this study is the first to conduct a thorough head‐to‐head comparison of EV isolation/enrichment methods from human stool specimens. We assessed the performance of each method using the following criteria: (1) particle recovery, (2) reproducibility, (3) purity, (4) protein yield/expression, and (5) RNA composition. This work has the potential to serve as the groundwork for future studies that use stool‐derived EV contents and high throughput ‐omics profiling to identify CRC biomarkers. Additionally, further examination of the separation of host‐derived and microbiome‐derived EVs may provide novel biological insights in CRC pathogenesis.

Our data confirmed the presence of EVs in all five isolation methods tested using transmission electron microscopy. Ultracentrifugation is often considered the gold standard for EV isolation; however, the UC‐P100k resulted in the lowest recovery rate (9%). The poor particle recovery with UC has also been observed with plasma using flow cytometry as analytical tool (Mastoridis et al., 2018; Tian et al., 2020). To improve particle recovery with UC, we performed six centrifugation scenarios and one method (single centrifugation at 80,000 x g for 2 h) led to higher particle recovery (∼30%) but it was also associated with abundant impurities based on TEM. High‐speed spins may cause damage to EV structural integrity (Baranyai et al., 2015; Linares et al., 2015; Monguió‐Tortajada et al., 2019) leading to reduced RNA and protein yield, which is likely the reason for no EV protein markers being detected with this method. The lower speed spin (P20k) resulted in better recovery (19%), but still underperformed when compared to both ExoQuick TC and Original (23% and 28%). Ultrafiltration and SEC had the best recovery rates overall (53% and 49%) when starting with 100ul of stool supernatant. However, when volume was increased to 500ul of stool supernatant, SEC had the highest recovery, and UF was extremely low. This indicates that UF may be susceptible to system saturation leading to poor efficiency. High particle yield is an important criterion for selecting the best EV separation method for downstream experiments for many biofluids due to limited volumes in a clinical setting. Fortunately, in the case of stool supernatant we have large volumes to work with. Specifically, when comparing biofluids, platelet free plasma (PFP) had ∼47 fold more particles than cell free urine, and stool supernatant has ∼5 fold more particles than PFP.

Lack of reproducible EV isolation methods has been a hindrance in EV research. We measured the reproducibility of particle recovery for each method, and the median coefficient of variation was less than 15%. Besides UC, all techniques require minimal handling and were performed according to the manufacturer's instructions. Despite UC being a commonly used EV isolation method, several studies have reported poor reproducibility (Tian et al., 2020; Vanderboom et al., 2021). For the current study, UC had similar reproducibility compared to the other methods. However, all UC experiments were conducted by a single operator, thus, exclude inter‐user variability in the assessment.

Purity is another selection criteria that should be considered when selecting the best EV isolation method for stool specimens. In the current manuscript, we examined visual contamination (TEM), non‐vesicular RNA contamination (enzymatic treatment) and protein contamination (particle count/protein yield ratio, western blot). SEC and UC‐P100k appeared to have the least visible contaminants by TEM. Both ExoQuick products had visual impurities which is in line with previous literature that concludes in addition to exosomes, nonexosomal proteins, immunoglobulins, viral particles, immune complexes, and other contaminants of overlapping size are found in the pellet from PEG‐based isolation procedures (Konoshenko et al., 2018; Li et al., 2017). For the ratio calculation, the presence of protein aggregates may contribute to particle count causing overestimation of purity. Ultrafiltration had the lowest ratio which indicates that it has the most soluble protein contamination, and the UC‐P100k had the leastsoluble protein contamination. Unlike with other biofluids such as plasma (albumin, lipoproteins) (Sódar et al., 2016) or urine (Tamm‐Horsfall glycoprotein‐THP) (Dhondt et al., 2020), there is no published research looking at soluble protein contaminants in stool. In an attempt to assess the presence of protein contaminants in isolated EVs, we measured the levels of chylomicrons using the surface marker Apolipoprotein B (ApoB) (Nakajima et al., 2014). Chylomicrons are low‐density lipoprotein particles sharing similar size and light scattering properties with EVs (Simonsen, 2017). For this reason, chylomicrons can be detected by flow cytometry (Sódar et al., 2016). We did not detect high levels of ApoB^+^ particles in stool supernatant compared to platelet‐free plasma which suggests that chylomicrons are not a major contaminant in stool from healthy donors. Additionally, we did not detect ApoB in the stool supernatant or purified samples by western blot. Calnexin is a protein associated with the endoplasmic reticulum that can be released by damaged cells and should not be present in purified EV samples (Théry et al., 2018). Calnexin was detected in stool supernatant but was not present in purified samples from any of the methods. Flagellin and GroEL have been identified in gram negative bacterial EVs (Alkandari et al., 2020; Hong et al., 2019; Piotrowska et al., 2020; Tulkens et al., 2020). Previous literature reported that flagellin and GroEL had higher levels detected in crude input EVs compared to purified EVs. Flagellin is a major subunit of flagella, which is considered an unwanted contaminant in EV preparation (Klimentová & Stulík, 2015). Additionally, another study exposed outer membrane vesicles (OMVs) to proteinase K, and flagellin was not detected by western blot, which further supports its location on the exterior of vesicles (Kunsmann et al., 2015). In the current study, flagellin was initially detected in UC‐P20k, EQ‐O, and EQ‐TC. When twice the amount of protein was loaded, it was also detected in three of the five SEC samples by western blot. The use of GroEL as a potential EV marker is inconclusive. It may form complexes with misfolded or damaged proteins that are being excreted or bind to extravesicle aggregates and lysed cells that stick to the outside of EVs (Hong et al., 2019). In the current study, it was detected in all methods except UC‐P100k. Further experiments are necessary to identify soluble proteins specific to stool that may contaminate EV preparations. A possible strategy could be mass spectrometry‐based proteomic profiling of SEC fractions enriched in soluble proteins (> F16) and EV rich fractions (F7‐F11) to determine the abundance and repertoire of secreted proteins using the human secretome database (Uhlén et al., 2019).

RNA biomarkers are of growing interest due to improvements in RNA extraction kits, library prep kits, and sequencing technologies over the years. Additionally, RNA can be quantified at low abundances with high sensitivity and specificity, and unlike DNA it provides dynamic insights into cellular states and regulatory processes. While several studies have examined the use of RNA in faeces as potential biomarkers for colorectal cancer (Gharib et al., 2021; Herring et al., 2021; Phua et al., 2014; Tarallo et al., 2019; Yau et al., 2016; Yau et al., 2014), none have focused on RNA within isolated EVs specifically. Chip‐based capillary electrophoresis (Agilent bioanalyzer) revealed that stool supernatant without EV isolation led to higher RNA yields compared to post‐isolation, but most of the fragments were smaller in size (40–60 nt). This data supports the idea that the vesicle serves as a barrier preventing RNase degradation. RNA fragment distribution analysis without enzymatic treatment revealed similar fragment sizes across the different isolation methods. Ultrafiltration and UC‐P100k were the methods with the highest and lowest RNA yield among samples, this correlates with the recovery efficiency data.

For EV biomarker discovery high purity separation of non‐vesicular RNA from EV derived RNA are critical for downstream transcriptomic technologies such as sequencing and RT‐qPCR. To further examine the presence of non‐vesicular RNA contamination we exposed EV fractions to proteinase K and RNase A prior to RNA extraction and compared the RNA yield to samples that had no enzymatic treatment prior to RNA extraction. The addition of proteinase k is necessary to expose RNAs in protein complexes to RNase activity (Théry et al., 2018). We selected UF and SEC samples for further enzymatic treatment because they had the highest recovery efficiency. Our data revealed that treatment with the enzyme combination resulted in a significant reduction in final RNA yield for UF and SEC samples. Ultrafiltration samples had more non‐vesicular RNA contamination compared to SEC. The higher RNA yield after enzymatic treatment among UF samples may be due to residual RNA that was not fragmented completely due to high concentrations of non‐vesicular RNA initially present in the samples. Based on the fragment size analysis, there was a decrease in small fragments (26–50 nt) among both UF and SEC samples post enzymatic treatment. Again, this is likely due to non‐vesicular RNA being shorter because of RNase degradation. After running UF samples on a small RNA bioanalyzer chip it was determined that the majority of small RNAs were miRNAs from outside of the vesicle. The SEC samples had considerably less non‐vesicular small RNA/miRNA contamination.

There is growing interest in understanding the role of the intestinal microbiome in human health. Studying this interaction may be especially important in the case of CRC since tumours more commonly develop in the distal colon and rectum, where 70% of the host's microorganisms reside (Ley et al., 2006). Extracellular vesicle release is an evolutionary conserved mechanism, and there is evidence that bacterial EVs (OMVs) from the host microbiome can enter the circulatory system and transfer biomolecules to mammalian host cells in distant organs (Bittel et al., 2021; Chronopoulos & Kalluri, 2020; Tulkens et al., 2020). Previous research has identified that both mice and human faeces contain significant amounts of EVs (Park et al., 2018). Enzyme linked immunosorbent assays (anti‐lipid A and anti‐lipoteichoic acid) and western blot (B actin) determined that the EVs were derived from both gram‐positive and negative bacteria, as well as from the host itself (Park et al., 2018). In the current study, we detected both human and bacterial EV proteins in stool specimens. As reported previously, ompA and LPS are bacterial EV protein markers (Tulkens et al., 2020). Using western blot, we detected ompA in all methods except UC‐P100k, and LPS was detected in one SEC sample. Additionally, ompA was also visible on the surface of EVs using immunogold labelling. Previously identified protein markers for human derived EVs were detected in all methods except UC‐P100k, these included: TSG101, CD63, and HSP70. To gain a better understanding of the proportion of bacteria and host derived RNA in stool EV preparations, RT‐qPCR assays were designed to amplify 16s rRNA and18s rRNA. When the RNA was pooled from all five methods, 16s rRNA was more abundant (1097x) than 18s rRNA. Kameli and others used a combination of ultrafiltration and SEC to isolate faecal membrane vesicles (Kameli et al., 2021). They implemented bead‐based flow cytometry to further characterize the abundance of gram positive (LTA), gram negative (omPA, LPS), and host (CD63/CD81/CD9) derived vesicles. They established that the vesicles labelled with bacterial markers showed higher positive signals compared to human EV markers which agrees with our RNA data (Kameli et al., 2021).

Recently, there have been studies that investigated the separation of bacterial and host derived EVs in stool. Park and others (2018) first proposed that the size of the EVs may be related to the origin of the EVs, with bacterial EVs being relatively smaller than host cell derived EVs through their use of germ‐free mice (Park et al., 2018). Tulkens and others (2020) further developed a method to separate and characterize bacterial EVs from host derived eukaryotic EVs in stool using ultrafiltration (> 0.22 uM) followed by bottom‐up density gradient centrifugation (bacterial EVs have higher density than host EVs) and SEC (Tulkens et al., 2020). Overall, we used similar techniques for EV isolation, but they took additional steps with longer processing times to further separate out bacterial EVs. Their proteomic analysis on bacterial EVs identified outer membrane proteins that may serve as critical surface proteins that allow for the separation of subpopulations using immuno‐affinity capture. Further research is necessary to investigate these surface markers for the development of immunocapture protocols. Ultimately, the depletion or enrichment of specific EVs populations would likely promote the identification of CRC biomarkers with increased sensitivity.

We recognize there are some limitations associated with the current study. While we assessed purity using multiple techniques (electron microscopy, western blot, particle to protein ratio), further analysis using proteomics will give a more detailed profile of protein contaminants. Additionally, the proteinase K added prior to RNase A ensures degradation of RNPs, but it is unclear if proteinase K also alters the surface of EVs resulting in RNase activity within vesicles (Qadir et al., 2018). However, without the addition of proteinase K, non‐vesicular RNA bound to RNPs would heavily contaminate vesicular RNA composition. PCR inhibitors are known to be present in stool, which may influence RT‐qPCR results (Wilde et al., 1990), the addition of PVPP and the use of diluted cDNA should mitigate these effects. Lastly, for the RT‐qPCR assays we pooled RNA from all methods to get a better understanding of the ratio of bacterial and host‐derived RNA. We are aware that it would have been more informative to run the assays on each method separately in order to determine if certain methods preferentially enrich for bacterial versus host derived EVs. However, we were unable to identify multiple reference genes for normalization purposes, which is a requirement according to MIQE guidelines (Bustin et al., 2009).

In conclusion, we confirmed the presence of bacterial and host derived EVs by western blot and immunoelectron microscopy. We determined that bacterial rRNA is more abundant than human rRNA based on our RT‐qPCR data in stool. Overall, ultrafiltration had the highest EV recovery, RNA, and protein yield, while ultracentrifugation performed the worst. Among the five techniques tested, SEC appears to be the most suitable EV isolation method for stool supernatant based on recovery and purity parameters. In the future, this method can be used to investigate stool‐derived EVs as a potential source of biomarkers to further enhance the detection of advanced precancerous lesions before they turn cancerous. Metagenomic, transcriptomic, and proteomic technologies offer multiple approaches for biomarker discovery that should be investigated further.

## DISCLOSURE STATEMENT

William R. Taylor and John B. Kisiel are inventors of Mayo Clinic intellectual property, licensed by Exact Sciences (Madison, WI, USA), from which royalties may be paid to Mayo Clinic. In addition to grants NCI CA214679 and CA 241164, John B. Kisiel receives funding from a sponsored research agreement between Mayo Clinic and Exact Sciences.

## ETHICAL STATEMENT

This work was approved by the Mayo Clinic Institutional Review Board with informed patient consent under IRB 18–008752 and 19–011292.

## Supporting information

Supporting information

## Data Availability

Data used to in this work will be made available upon request and is conditional to approval from the Mayo Clinic Institutional Review Board.
