# Supplementary material for: Assessment of extracellular vesicle isolation methods from human stool supernatant
Source: J Extracell Vesicles. 2022 Apr 5;11(4):e12208. doi: 10.1002/jev2.12208 (PMC8980777; doi:10.1002/jev2.12208)
Supplement: Supplementary file 1 — Supporting information [file JEV2-11-e12208-s001.docx]

**Supplemental** **Figure Legends:**

**Figure S1. Evaluation of six ultracentrifugation (UC) protocols for EV recovery (nFCM) and purity**​**(TEM).**(A) 100 microliters of stool supernatant (n=5 patients) underwent EV isolation using the six UC ​scenarios, (B) Particle recovery from the pellet that was generated from the high-speed spin(s) (C) Total​ particle recovery from the low (P20k) and high- speed spin (s), Mean ± SEM, One-way ANOVA test, **p*< 0.05.

**Figure S2. Assessment of purity of UC EV preparations from human stool specimens by electron microscopy**. Representative EM pictures showing EVs isolated from four ultracentrifugation scenarios. Lower magnification (15kx) shows a wider field of view and a better representation of contaminants in samples. White arrows at higher magnification (100kx) indicate individual EVs. Scenario D (100,000 x g 2hr, twice) appears to be the purest method with the least visible contaminants at both magnifications. No lipoprotein contamination was observed with any of the methods.

**Figure S3. Sensitive detection of submicron particles by nFCM.**(A) Representative flow cytometry scatterplots and graphs of levels of ApoB-positive lipoproteins measured in platelet-free plasma (n=5) and stool supernatants (n=5), Non-parametric *t-*test, ***p*< 0.01 (B) Western blot showing the absence of ApoB in all methods, with CD63 as a positive EV marker control (C) Scatterplots and histograms showing resolution of a population of polystyrene and silica beads with a diameter ranging from 110 nm to 1,300 nm (D) Scatterplot showing nFCM detection of a polystyrene bead population of 80 nm. By applying Mie Theory modeling, side scatter intensities can be converted in diameter. In our nFCM, a polystyrene bead population of 80 nm scatter as much light as an EV of 158 nm (RI core= 1.38; RI shell= 1.48; shell thickness= 4 nm).

**Figure S4. nFCM-based comparative analysis of EV isolation/enrichment methods for particle recovery from 5 healthy human stool specimens.** (A) total particle count pre-isolation and following low-speed (P20k) and high-speed centrifugation (P100k), total particle count of the concentrate from ultrafiltration, total particle count for fractions 7-11 from size exclusion chromatography, and total particle count from pellets formed after ExoQuick incubation and centrifuge spin.

**Figure S5. Percent particle recovery for patient replicates within each isolation method.**There appears to be limited variability within patient replicates for each method. The source of variability within each isolation method likely originates from differences in stool composition among the patients.

**Figure S6. nFCM-based analysis of particle concentration in larger volume of supernatant and comparison in different biofluids.**​ (A) Total particle recovery from 500 microliters of stool supernatant (n=5 samples) processed with four isolation methods, Mean ± SEM, One-way ANOVA test, ***p*< 0.01, ****p*< 0.0005, *****p*< 0.0001 (B) Particle concentration in cell-free urine (n=5), platelet-free plasma (n=5) and stool supernatants (n=5), Mean ± SEM, One-way ANOVA test, **p*< 0.05, ***p*< 0.01.

**Figure S7. RNA yield and composition after proteinase k and RNase A treatment.**(A) Small RNA chip bioanalyzer results from a stool supernatant sample that underwent EV isolation by SEC or UF and was either treated with proteinase K/RNase A treatments or not prior to RNA extraction. (B) RNA 6000 pico chip bioanalyzer results from a stool supernatant sample that underwent EV isolation by SEC or UF and was either treated with proteinase K/RNase A treatments or not prior to RNA extraction.

**Figure S1.**

**A**


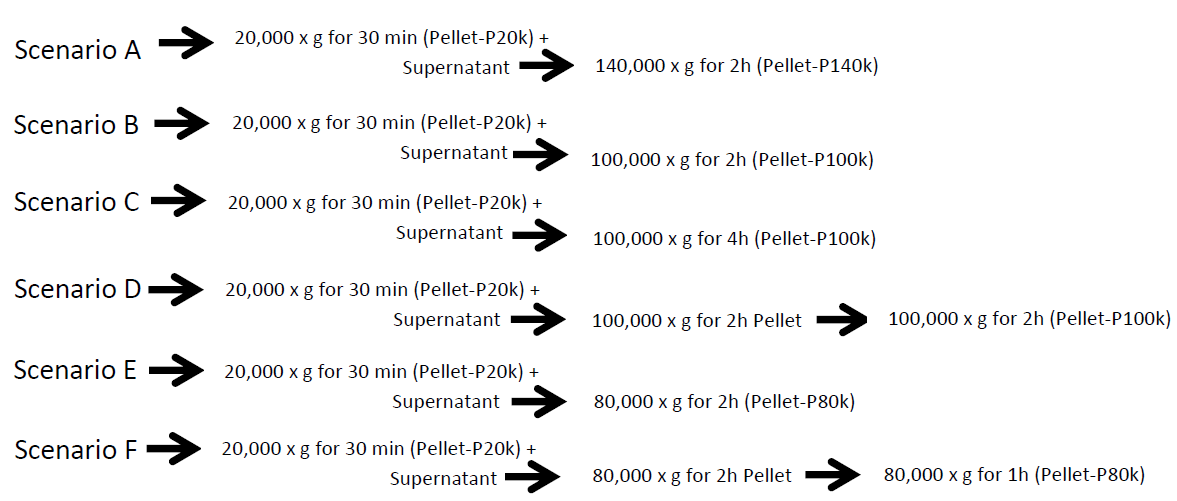


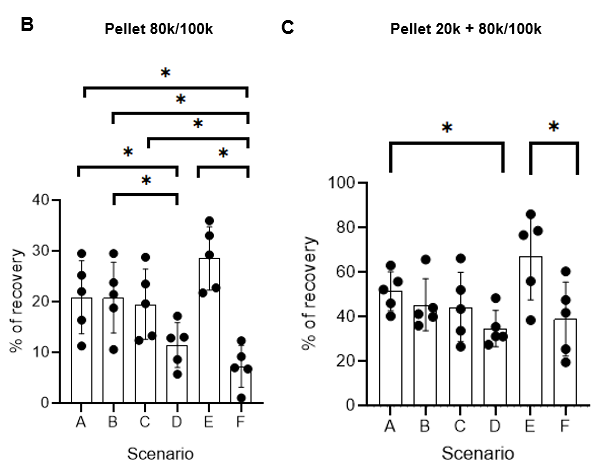


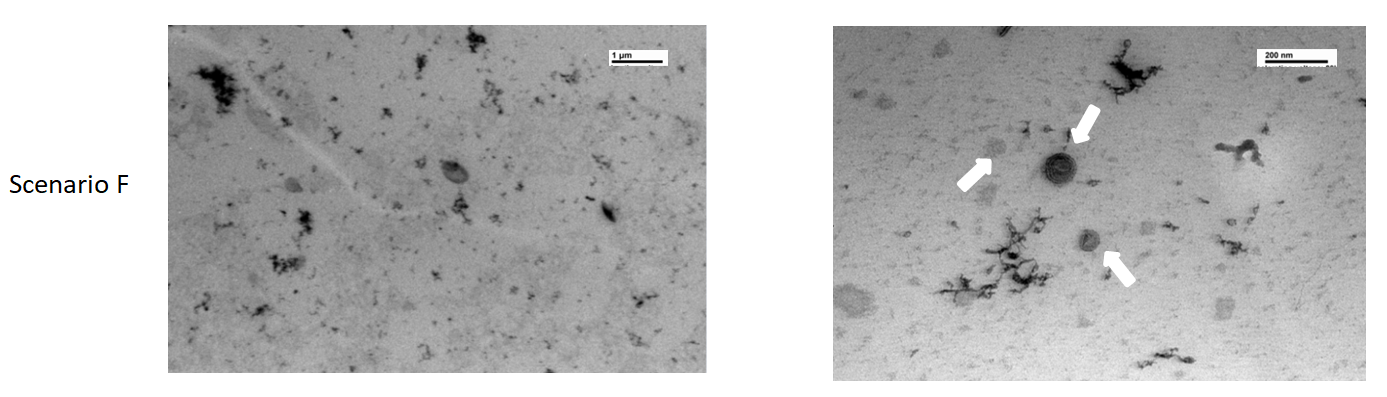

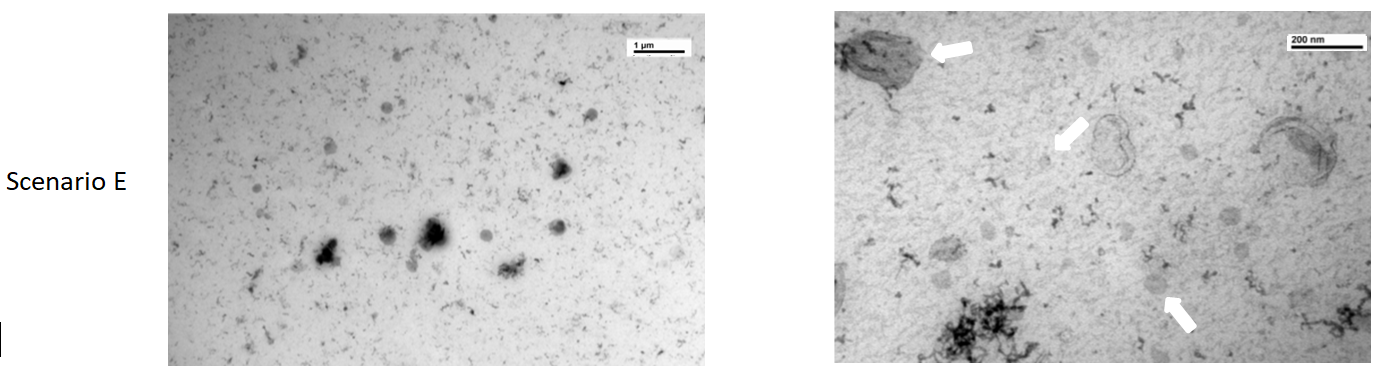

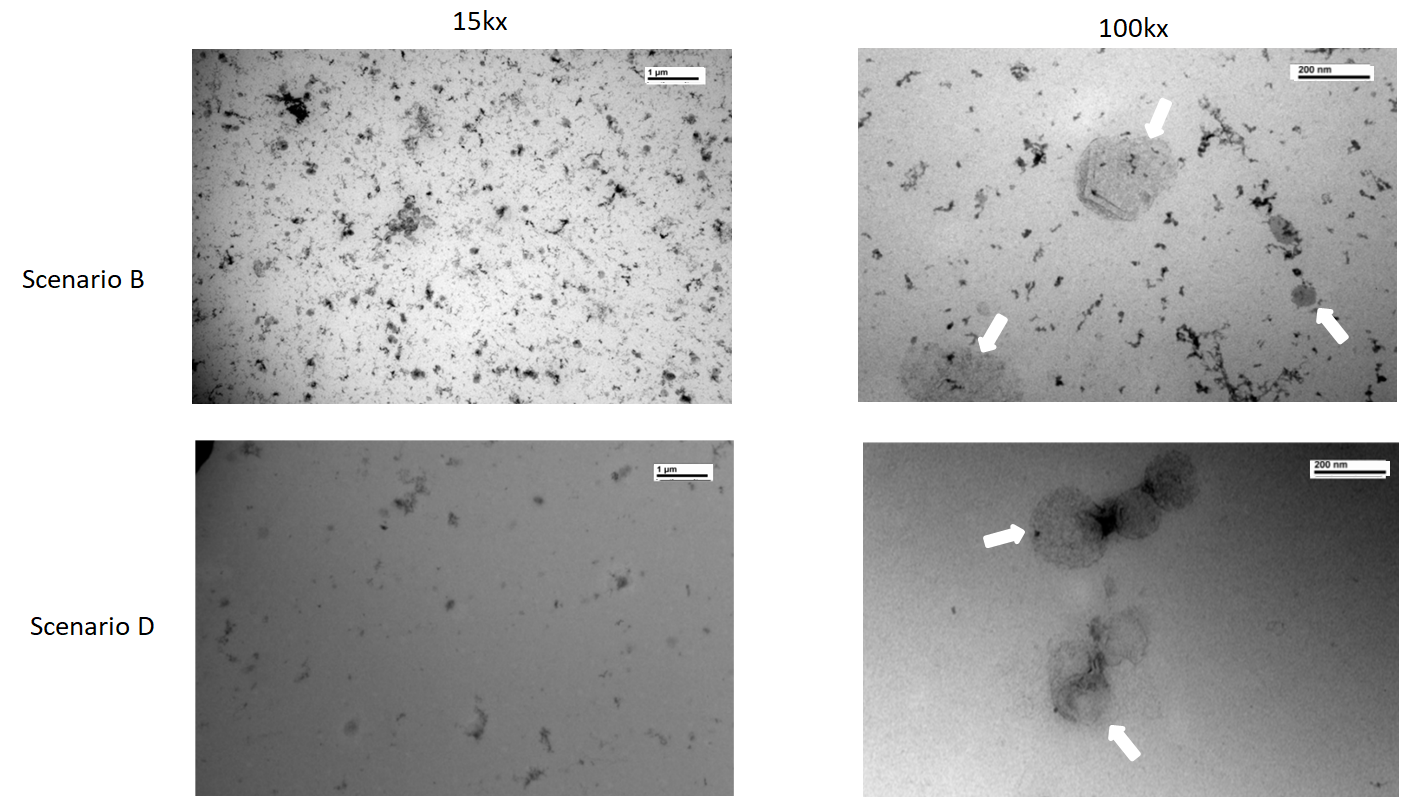
**Figure S2.**

**Figure S3.**

A


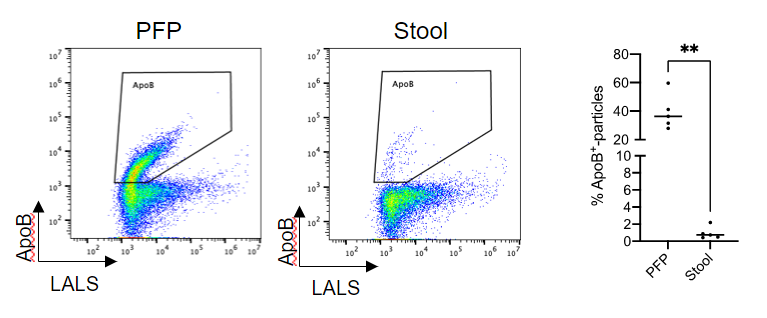


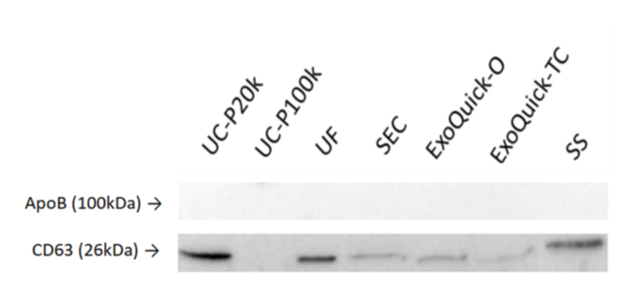


B


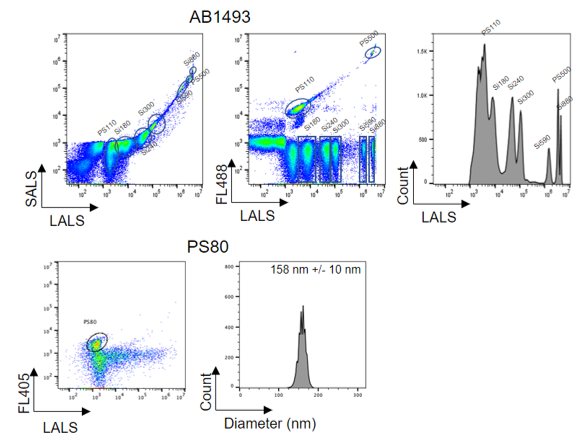


C

D

**Figure S4.**

**
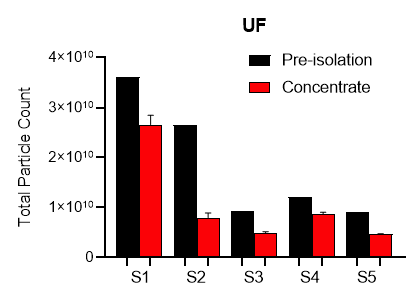
**
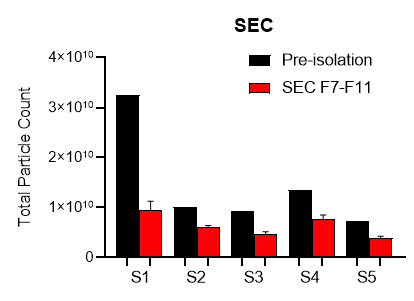


**
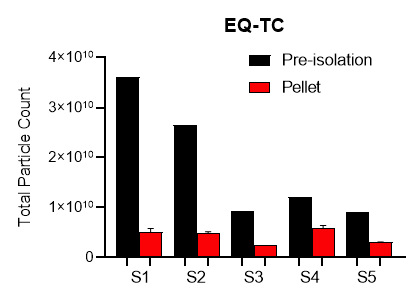

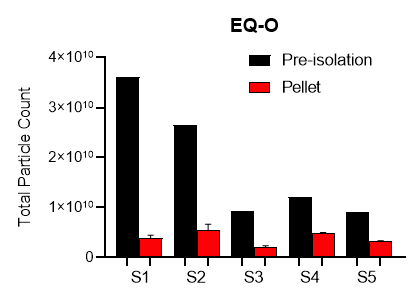
**

**
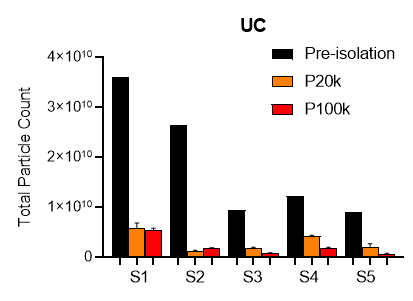
**


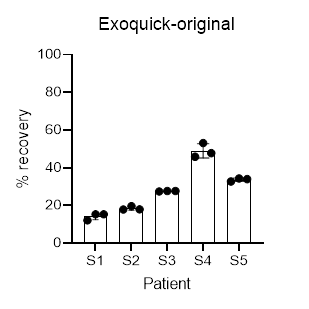
 **Figure S5.**

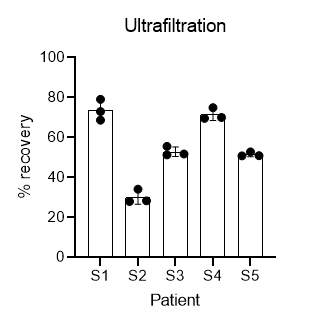

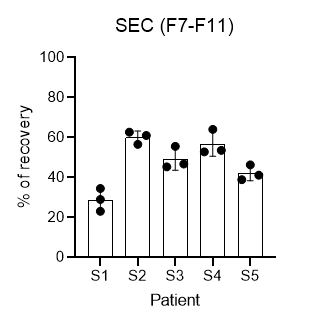


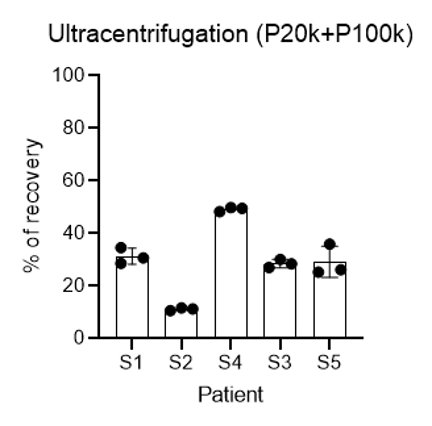


**Figure S6.**


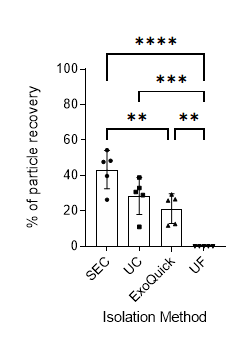


B

A


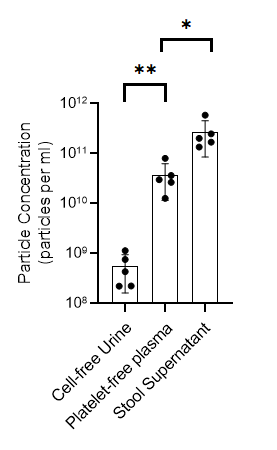


**Figure S7.**

A


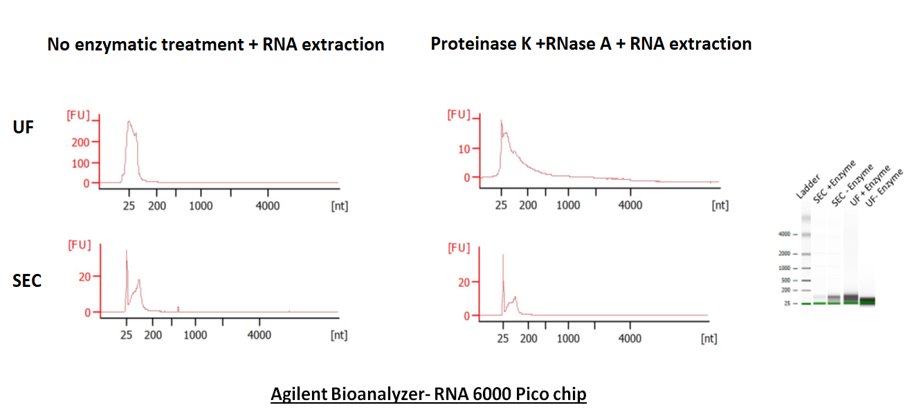

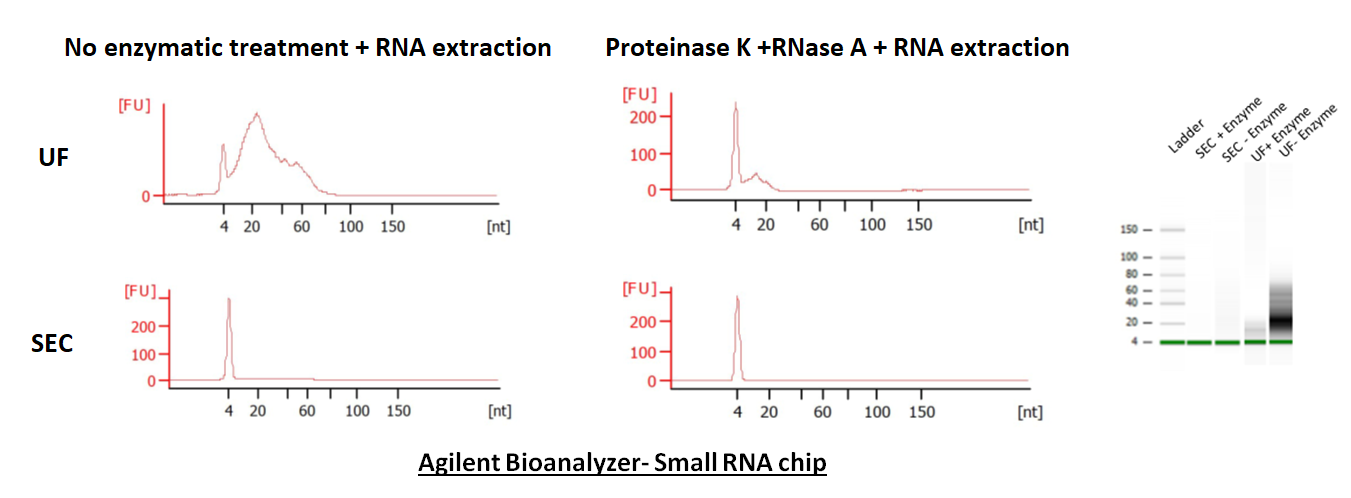


B
